# A Phenomenological Inquiry into the Contribution of Positive Emotions to L2 Teachers’ Work Engagement: Focusing on Iranian Teachers’ Insights and Lived Experiences

**DOI:** 10.11621/pir.2025.0301

**Published:** 2025-09-01

**Authors:** Ali Derakhshan, Mohammad Sadegh Taghizadeh

**Affiliations:** a Golestan University, Gorgan, Iran; b Allameh Tabataba’i University, Tehran, Iran

**Keywords:** L2 teachers, phenomenological inquiry, positive emotions, work engagement

## Abstract

**Background:**

Due to its prominence in the professional success of teachers, work engagement has gained increasing attention in educational research, particularly in language studies. While previous studies have investigated the contribution of various factors to language teachers’ work engagement, the role of emotional experiences, notably positive emotions, remains under-explored.

**Objective:**

To bridge this gap, the current research sought to examine the interplay between positive emotions and work engagement from the standpoints of Iranian L2 teachers. It was also aimed at pinpointing the particular positive emotions that most significantly contribute to the work engagement of L2 teachers.

**Design:**

Phenomenology as a qualitative research approach was employed to scrutinise the perceptions and lived experiences of participants. With the aid of maximum variation sampling, 37 L2 teachers were selected from diverse educational institutions in Iran. To delve into the participants’ perspectives and experiences, an open-ended questionnaire and a semi-structured interview were employed. Participants’ answers to the questionnaire and the follow-up interview were analysed through inductive thematic analysis.

**Results:**

The thematic analysis revealed that Iranian L2 teachers perceived a close and dynamic association between positive emotions and work engagement. The analysis further indicated that, according to Iranian L2 teachers, positive emotions such as enjoyment, contentment, passion, pride, and hope can significantly contribute to their work engagement.

**Conclusion:**

The study’s findings highlight the importance of positive emotions in promoting the work engagement of L2 teachers and suggest that educational administrators should prioritise creating a supportive working environment that cultivates positive emotional experiences.

## Introduction

Work engagement is a critical factor influencing the overall performance of individuals and their productivity in professional environments (Lee et al., 2024; Schaufeli, 2021). It refers to “one’s positive, fulfilling state of mind characterised by vigour, dedication, and absorption” (Schaufeli et al., 2009, p. 894). As an indicator of professional commitment and vitality, work engagement reflects the extent to which individuals are ‘absorbed in’, ‘enthusiastic about’, and ‘committed to’ their work (Schaufeli, 2013; Zhi & Derakhshan, 2024). In this study, work engagement is defined as second and foreign language (L2) teachers’ enduring, positive psychological states, characterised by their enthusiasm, dedication, and deep involvement in professional responsibilities. In instructional settings, notably second and foreign language (L2) teaching contexts, work engagement becomes even more crucial because it directly influences the quality of instruction, student motivation, and learning outcomes (Al-Obaydi et al., 2023; Derakhshan et al., 2023; Estaji & Taghizadeh, 2024). Therefore, fostering and sustaining the work engagement of L2 teachers is essential for enhancing instructional quality, maintaining academic motivation, and promoting learning outcomes.

Given the value of work engagement in L2 teaching contexts, factors that underlie the work engagement of L2 teachers need to be disclosed. In response to this need, numerous language researchers have probed the role of various external and internal factors in L2 teachers’ work engagement (*e.g.*, Greenier et al., 2021; Wang et al., 2024; Zhi et al., 2024). However, existing studies have primarily focused on the role of external factors such as administrative support, teaching resources, and instructional environment (Addimando, 2019; Derakhshan et al., 2025; Li & Xu, 2024). While these studies have offered valuable insights into the determinants of teacher work engagement, they have largely neglected the contributions of internal elements, particularly positive and negative emotions. To narrow this lacuna, the current research intends to examine the potential contribution of positive emotions to the work engagement of L2 teachers.

Emotions are integral to instructional contexts, shaping both teaching practices and learning outcomes (Blake & Dewaele, 2023; Derakhshan & Zhang, 2024). Positive emotions, in particular, have been shown to enhance creativity, problem-solving capacities, and interpersonal relationships—all of which are essential for effective teaching (Dewaele, 2018; Xie & Jiang, 2021). These emotions have also been found to foster a supportive and motivating teaching atmosphere, which is crucial for teachers’ sustained engagement (Burić & Macuka, 2018; Fathi et al., 2024; Xiao et al., 2022). In this study, positive emotions refer to affective states such as joy, enthusiasm, gratitude, and pride that L2 teachers experience in their professional roles.

From a theoretical perspective, Fredrickson’s “broaden-and-build theory” (BBT) of positive emotions provides a compelling framework for understanding their role in teacher work engagement. According to this theory, positive emotions broaden an individual’s thought-action repertoires and build lasting personal and interpersonal resources that are critical to their engagement with their environments (Fredrickson, 2004, 2013). For L2 teachers, this means that experiencing positive emotions can promote their creativity, resilience, and ability to connect with colleagues and students, ultimately contributing to sustained work engagement (Qu et al., 2025; Wang & Pan, 2023; Zhi & Wang, 2025).

By applying this theoretical lens, this research seeks to identify the role of positive emotions in the work engagement of L2 teachers. Through a phenomenological approach, the research attempts to capture the lived experiences and subjective perceptions of Iranian L2 teachers, providing nuanced, rich insights into the interplay between positive emotions and teacher work engagement. By focusing on the affective predictors of teacher engagement, this study not only addresses the existing gaps in the literature but also offers practical implications for supporting teachers’ sustained engagement in L2 classrooms. More specifically, this qualitative research aims to address the following questions:

- How do Iranian teachers perceive the interplay between positive emotions and work engagement in L2 instructional contexts?
- From the viewpoint of Iranian teachers, what positive emotions contribute to work engagement in L2 instructional contexts?

## Methods

This qualitative study adopted a phenomenological approach to explore Iranian L2 teachers’ perceptions and lived experiences of the interplay between positive emotions and work engagement. Phenomenology, developed by Heidegger (2005), provides a suitable framework for examining how participants interpret and make sense of the phenomenon under study.

### Participants

The study involved 37 Iranian L2 teachers, selected through maximum variation sampling from diverse educational settings, including schools, universities, and language institutes. We selected this non-probability sampling technique to ensure a broad representation of diverse L2 teachers, helping us to capture a wide array of experiences and perspectives. The technique allowed for the inclusion of L2 teachers with varying personal and professional characteristics, including age, gender, academic major, academic degree, and teaching experience (**Table 1**).

**Table 1:** Demographic information

		Number	Percent
Gender	Male	21	56.8
	Female	16	43.2
Age	25–30	18	48.6
	31–36	9	24.3
	37+	10	27.1
Teaching Experience	5–10	17	45.9
	11–16	8	21.6
	17+	12	32.5
Academic Major	Teaching English as a Foreign Language (TEFL)	23	62.2
	English Language and Literature	9	24.3
	English Language Translation	5	13.5
Academic Degree	Master of Arts (MA)	15	40.5
	Doctor of Philosophy (Ph.D.)	22	59.5
Educational Context	Language Institute	12	32.5
	School	7	18.9
	University	18	48.6
Total		37	100

Prior to their involvement in the study, all participants signed a consent letter to affirm that they were fully informed about the purpose, procedure, and voluntary nature of this research.

### Data Collection Instruments

#### Open-ended Questionnaire

The study employed an open-ended questionnaire to delve into the unique experiences and insights of Iranian L2 teachers regarding the contribution of positive emotions to their work engagement. This instrument was chosen for its capacity to capture rich, detailed, and reflective responses from participants while offering them the flexibility to express their thoughts without constraints (Brown, 2009). The questionnaire, which consisted of two open-ended questions, was reviewed by two qualitative research experts and refined based on their suggestions and feedback. A pilot test was then conducted with a smaller group of L2 teachers to fine-tune the wording and structure of the questions, ensuring that they were clear and conducive to eliciting meaningful responses.

#### Semi-structured Interview

Semi-structured interviews were conducted to complement the data collected through the open-ended questionnaire, providing deeper insights into how positive emotions contribute to their work engagement. This flexible yet guided approach enabled an in-depth exploration of the perspectives of participants while allowing room for their spontaneous insights (Richards, 2009). The interview guide included two broad, open-ended questions designed to elicit reflective narratives from interviewees. Some follow-up questions were also used to clarify responses and encourage the interviewees to share detailed accounts.

### Procedure

The study’s data collection conducted in two well-defined phases. In the first phase, an open-ended questionnaire containing two carefully designed questions was distributed to participants (*N* = 37). The questionnaire was shared electronically to accommodate the schedules of participants and their preferences, allowing them adequate time to provide detailed and thoughtful answers. In the second phase, semi-structured interviews were conducted with a subset of participants (*N* = 14) who wanted to further elaborate on their emotional experiences. The interviews were scheduled at mutually convenient times and conducted either in person or virtually, depending on the preference of the interviewee. Each interview, lasting around twenty minutes, was audio-recorded with the consent of the interviewees to ensure accurate transcription and thorough analysis. This two-phase procedure ensured a comprehensive understanding of the role of positive emotions in their work engagement. The open-ended questionnaire provided broad insights and the semi-structured interview gave deeper, more nuanced perspectives.

### Data Analysis

The data obtained from the open-ended questionnaires and semi-structured interviews were analysed using thematic analysis, a widely recognised and flexible analytical method that allows for the identification of patterns or themes within textual data (Braun & Clarke, 2006). This method was selected for its capacity to uncover both explicit and implicit meanings embedded in the responses, offering a nuanced understanding of the contribution of positive emotions to the work engagement of the participants.

The analysis began with a thorough reading of the transcriptions from the interviews and the open-ended questionnaire responses, ensuring familiarity with the data and providing an initial sense of the participants’ perspectives. This initial phase allowed the researchers to identify potential areas of interest and to begin forming preliminary ideas regarding the content of the data. Subsequently, the data were coded inductively, meaning that codes were derived directly from the data rather than being predetermined. This approach ensured that the analysis was data-driven, with the themes emerging naturally from the participants’ responses. The codes were then organised into broader categories, which were further refined into key themes related to the interplay between positive emotions and teacher work engagement. Throughout the process, the researchers repeatedly checked the consistency and relevance of the emerging themes by cross-referencing the codes with the original data. This iterative process ensured that the analysis remained grounded in the voices of the participants. MAXQDA soft ware (v. 2024) was used to assist with the coding and categorisation process, providing a systematic approach to managing the obtained data. The soft ware facilitated the organisation of codes, retrieval of relevant excerpts, and comparison of themes across participants. Finally, the identified themes were interpreted in relation to the research questions, and key insights were drawn regarding the interaction of positive emotions and teacher work engagement. Following the thematic analysis, through Cohen’s Kappa (к), the “intercoder reliability” (ICR) was assessed to ensure the consistency and agreement between the researchers involved in the coding process. The Cohen’s Kappa value was 0.94, indicating a strong level of consistency in the coding process.

To ensure the rigour and quality of this study, the key principles of good qualitative research were upheld (Nassaji, 2020). Specifically, credibility was maintained through the use of member checking, where participants were asked to confirm the accuracy of the interpretations drawn from their perceptions and lived experiences. Trustworthiness was bolstered by the collaborative nature of the analysis, with two researchers independently coding the data to ensure consistency and reduce individual bias. An audit trail was further established by keeping detailed records of the data collection, coding process, and theme development, which allowed for transparency and accountability in the research process. Transferability was also supported by providing rich, thick descriptions of the participants’ experiences and the context of the study. Additionally, triangulation was achieved by combining data from the open-ended questionnaire and semi-structured interviews, further enhancing the study’s depth and validity.

## Results

This section presents the findings of the study, organised into two distinct parts in response to the research questions. First, the perceptions of Iranian teachers regarding the interplay between positive emotions and work engagement in L2 instructional contexts are discussed. This part explores how Iranian teachers view the relationship between their emotional experiences and their overall engagement within L2 classrooms. The second part identifies the specific positive emotions that, from the Iranian teachers’ perspectives, contribute most significantly to their work engagement. This part highlights the key emotional experiences that enhance L2 teachers’ professional engagement and long-term commitment to their teaching roles.

### Interplay between L2 Teachers’ Positive Emotions and Work Engagement

The analysis revealed a strong, dynamic relationship between positive emotions and work engagement amongst Iranian L2 teachers. Participants consistently described how positive emotional experiences, such as enjoyment, pride, passion, hope, and contentment, enhance their professional engagement by fostering their motivation, energy, and a deeper sense of commitment to teaching roles. Specifically, participants reported that experiencing positive emotions increases their enthusiasm for teaching and encourages them to invest greater effort into their instructional practices. One teacher explained, *“When I feel happy and appreciated in my classroom, I find myself wanting to do more, to create more engaging lessons, and to connect more with my students”* (Participant 6). Moreover, a teacher stated, *“When I feel proud of my personal progress, I feel more energised and determined to put more effort into my lessons”* (Participant 11). Further illustrating this connection, another teacher reflected, *“On days when I feel satisfied with my teaching practices, I find myself being more energetic and willing to go the extra mile, even after a long day”* (Participant 17).

Positive emotions were also perceived to play a crucial role in sustaining the work engagement of the L2 teachers by enhancing their resilience and emotion regulation ability. Several teachers noted that positive emotions like hope and contentment provide them with a sense of purpose and meaning, which supports their long-term commitment to the profession. For example, one teacher shared, *“Even when I face educational adversities, my sense of hope keeps me motivated and engaged”* (Participant 8). Another teacher echoed this sentiment, stating, *“Hope gives me the strength to persist, even when things don’t go as planned. It keeps me focused on the positive aspects of my job that are essential for maintaining long-term professional engagement”* (Participant 24). Another teacher emphasised, *“When I feel content in my work, it reminds me of why I chose teaching in the first place, and that drives me to keep going even on tough days”* (Participant 29). Collectively, these reflections underscored that those positive emotions not only energise L2 teachers but also help them to maintain their focus, energy, and effort, even in times of challenges or adversities.

### Positive Emotions Contributing to the Work Engagement of L2 Teachers

**Figure 1.**
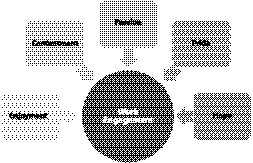
Positive emotions contributing to L2 teachers’ work engagement

The findings revealed that Iranian L2 teachers identified five key positive emotions— *enjoyment*, *contentment*, *passion*, *pride*, and *hope*—as the most significant contributors to their work engagement (*Figure 1*).

### Enjoyment

Enjoyment emerged as the most influential positive emotion driving work engagement among L2 teachers. Iranian teachers highlighted that experiencing enjoyment in their teaching roles energises them and increases their engagement levels within L2 instructional contexts. One teacher explained, *“When I enjoy teaching a lesson, I feel more connected to my profession, and it makes me want to dedicate more time and energy to my responsibilities”* (Participant 2). Another teacher stated, *“I feel more engaged when I genuinely enjoy the classroom atmosphere. It helps me maintain my enthusiasm throughout the lesson and motivates me to be more active”* (Participant 7).

### Contentment

Contentment was also identified as a crucial positive emotion contributing to L2 teachers’ work engagement. Teachers reported that feeling content with their work environment, student relationships, and professional achievements enhances their emotional well-being and reinforces their commitment to teaching. One teacher noted, *“When I feel content with my teaching environment and students’ progress, I am more focused and dedicated to my profession”* (Participant 4). Another teacher shared, *“Contentment gives me a sense of fulfillment, and it encourages me to invest much more effort in accomplishing my responsibilities”* (Participant 22).

### Passion

Passion was also reported as a strong, positive emotion that significantly enhances teachers’ work engagement in L2 classrooms. Iranian L2 teachers described how their deep passion for teaching fuels their professional engagement and inspires them to invest extra effort in their instructional practices. One teacher expressed, *“I am deeply passionate about teaching English, and this strong desire makes me remain engaged and motivated”* (Participant 16). Another teacher remarked, *“My passion for seeing my students grow inspires me to put more time and energy into my teaching duties”* (Participant 20).

### Pride

Pride was perceived as another important emotion positively influencing L2 teachers’ work engagement. Iranian teachers explained that feeling proud of their students’ achievements and their own teaching accomplishments boosted their confidence and inspired them to continue striving for excellence. One teacher stated, *“When I see my students succeed and feel proud of their progress, it motivates me to work even harder”* (Participant 5). Another teacher shared, *“Feeling proud of my teaching achievements inspires me and makes me more committed to my work”* (Participant 18).

### Hope

Hope was also highlighted as another positive emotion that can lead to increased teacher engagement. Iranian L2 teachers reported that being hopeful about their students’ future success and their own professional growth encourages them to stay motivated and engaged. One teacher noted, *“Hope for my students’ success gives me a sense of purpose. It keeps me engaged even when I encounter setbacks”* (Participant 13). Another teacher explained, *“Hope inspires me to look beyond immediate diffculties and stay focused on long-term goals, which helps me maintain my commitment to teaching”* (Participant 30).

Overall, according to Iranian teachers, positive emotions like enjoyment, contentment, passion, pride, and hope play a pivotal role in fostering and sustaining L2 teachers’ work engagement. These positive emotions increase teachers’ motivation, improve their emotional resilience, and strengthen their professional commitment, enabling them to maintain high levels of engagement in L2 instructional contexts.

## Discussion

This phenomenological study set out to investigate Iranian teachers’ experiences and perspectives on the contribution of positive emotions to the work engagement of L2 teachers. Specifically, it was aimed at understanding how Iranian teachers perceive the interplay between positive emotions and work engagement within L2 teaching environments. The study also sought to identify the specific positive emotions that underlie L2 teachers’ work engagement. Thematic analysis of the participants’ perceptions and lived experiences identified a close, dynamic relationship between L2 teachers’ positive emotions and work engagement. Additionally, enjoyment, contentment, passion, pride, and hope emerged as the most significant positive emotions driving the work engagement of L2 teachers.

The perceived association between positive emotions and teacher work engagement can be logically explained through the BBT. This theory posits that positive emotions expand an individual’s immediate thought-action repertoires, leading to the development of enduring emotional, social, and psychological resources (Fredrickson, 2004). These enduring personal resources, in turn, serve to improve an individual’s engagement with their environments (Fredrickson, 2013). Consistent with this perspective, the study’s findings demonstrated that the positive emotions of L2 teachers can boost and maintain their engagement in instructional environments by fostering their personal resources, such as resilience and emotion regulation ability. This aligns with the outcomes of prior studies emphasising the critical role of positive emotions in promoting the professional engagement of teachers (Azari Noughabi et al., 2022; Burić & Macuka, 2018; Yang et al., 2022).

In this study, enjoyment stood out as the most significant positive emotion contributing to the work engagement of L2 teachers. This implies that L2 teachers who find joy in their profession are more likely to engage actively in teaching contexts. This finding corroborates those of Fathi et al. (2024) and Xiao et al. (2022), who discovered that teaching enjoyment plays a pivotal role in fostering the work engagement of language teachers. Contentment was another positive emotion that emerged as a significant contributor to L2 teachers’ work engagement. According to this finding, L2 teachers who experience happiness and satisfaction in instructional settings tend to allocate more time to their job-related responsibilities. This result supports those of Antoniou et al. (2024) and Timms and Brough (2013), who reported that job satisfaction can contribute to increased teacher engagement. Passion was also identified as a key positive emotion in this study, significantly contributing to L2 teachers’ engagement at work. This suggests that L2 teachers who are passionate about their professional tasks and responsibilities tend to dedicate more time and energy to fulfilling them. This outcome is consistent with those of Trépanier et al. (2014) and Uslukaya et al. (2024), who discovered that teachers’ passion for teaching triggers them to actively engage in instructional contexts. Pride was also recognised as another crucial emotion that underlies teachers’ active engagement in L2 teaching environments. This indicates that the deep pleasure that L2 teachers derive from their own or their students’ accomplishments motivates them to regularly engage in teaching contexts. This result echoes Burić and Macuka’s (2018) outcomes, which demonstrated that positive emotions, including pride, can result in enhanced teacher engagement. Finally, the study identified hope as another important positive emotion that is essential to teachers’ sustained engagement in L2 classrooms. This finding mirrors that of Ugwu and Amazue (2014), who found that feeling of hope encourages teachers to remain engaged in their profession.

## Conclusion

The current research tried to identify the role of emotional experiences, notably positive emotions, in the work engagement of L2 teachers. The findings revealed that positive emotions— namely enjoyment, contentment, passion, pride, and hope— can enhance and sustain L2 teachers’ engagement by building enduring personal resources. From a theoretical perspective, these findings enrich the expanding body of research on the emotional foundations of L2 teachers’ work engagement by offering empirical support for the contribution of positive emotions to teacher engagement. This study highlights how emotions like enjoyment, contentment, passion, pride, and hope actively shape and sustain L2 teachers’ commitment and involvement in their professional roles, thus deepening our understanding of the emotional dynamics underlying teacher engagement. The study further supports and extends Fredrickson’s (2001) BBT, illustrating how positive emotions can broaden and build teachers’ emotional and psychological resources, which in turn promote their engagement within instructional environments.

From a practical standpoint, the findings have significant implications for educational administrators and teacher trainers. It is essential for educational administrators to create emotionally supportive environments that nurture positive emotions among teachers. According to the study’s outcomes, creating an emotionally supportive climate where teachers can experience positive emotions like enjoyment, contentment, and hope triggers them to put more time and energy into their professional tasks. Additionally, teacher training programmes could benefit from integrating resilience and emotion regulation trainings, which empower teachers to effectively manage their emotional experiences and remain engaged in their profession, regardless of instructional adversities.

## Limitations

Although this study offers valuable insights, it suffers from several limitations that need to be considered by future investigations. First, the study was conducted solely in Iranian L2 classrooms, which may restrict the applicability of the findings to other cultural or educational settings. Future studies could replicate this investigation with teachers from diverse cultural backgrounds to examine whether similar emotional dynamics influence teacher engagement across different educational systems. Second, the use of phenomenology as a qualitative research approach provided in-depth insights into teachers’ subjective perceptions and lived experiences but may not capture the broader prevalence or intensity of positive emotions that underlie work engagement. Future research could adopt a mixed-methods design, combining qualitative and quantitative approaches to provide a more comprehensive understanding of the emotional factors driving teachers’ work engagement. The third limitation of this research lies in its reliance on self-reported data, which is subject to personal bias and may not fully capture the complex interplay between positive emotions and teacher work engagement. By incorporating observational methods, future research could obtain more nuanced, in-depth data on the interactions of teachers’ emotional experiences and work engagement.
